# Colon Cancer-Upregulated Long Non-Coding RNA lincDUSP Regulates Cell Cycle Genes and Potentiates Resistance to Apoptosis

**DOI:** 10.1038/s41598-018-25530-5

**Published:** 2018-05-09

**Authors:** Megan E. Forrest, Alina Saiakhova, Lydia Beard, David A. Buchner, Peter C. Scacheri, Thomas LaFramboise, Sanford Markowitz, Ahmad M. Khalil

**Affiliations:** 1 Department of Genetics and Genome Sciences, Cleveland, OH 44106 USA; 2 Case Comprehensive Cancer Center, Cleveland, OH 44106 USA; 3 Department of Biochemistry, Case Western Reserve University, Cleveland, OH 44106 USA

## Abstract

Long non-coding RNAs (lncRNAs) are frequently dysregulated in many human cancers. We sought to identify candidate oncogenic lncRNAs in human colon tumors by utilizing RNA sequencing data from 22 colon tumors and 22 adjacent normal colon samples from The Cancer Genome Atlas (TCGA). The analysis led to the identification of ~200 differentially expressed lncRNAs. Validation in an independent cohort of normal colon and patient-derived colon cancer cell lines identified a novel lncRNA, lincDUSP, as a potential candidate oncogene. Knockdown of lincDUSP in patient-derived colon tumor cell lines resulted in significantly decreased cell proliferation and clonogenic potential, and increased susceptibility to apoptosis. The knockdown of lincDUSP affects the expression of ~800 genes, and NCI pathway analysis showed enrichment of DNA damage response and cell cycle control pathways. Further, identification of lincDUSP chromatin occupancy sites by ChIRP-Seq demonstrated association with genes involved in the replication-associated DNA damage response and cell cycle control. Consistent with these findings, lincDUSP knockdown in colon tumor cell lines increased both the accumulation of cells in early S-phase and γH2AX foci formation, indicating increased DNA damage response induction. Taken together, these results demonstrate a key role of lincDUSP in the regulation of important pathways in colon cancer.

## Introduction

Despite improvements in diagnostic and therapeutic strategies, colon cancer remains the third leading cause of cancer-related death in the United States, largely due to frequent treatment failure and recurrence^1,2^. While extensive studies of colon tumors have characterized protein-coding genes and epigenetic changes involved in tumor initiation and progression, the identification of regulatory RNAs, including microRNAs (miRNAs) and long non-coding RNAs (lncRNAs), has added a previously unrealized level of complexity to the molecular landscape of colon cancer^3,4^. LncRNAs are broadly categorized as transcripts >200 nt in length that are polyadenylated and spliced similar to mRNA transcripts, but have no protein-coding capacity^5–7^. Many lncRNAs show cell-type and developmental-stage specific expression, suggesting key roles in cell identity and tissue organization. Also, some lncRNAs become dysregulated in human disease, including cancer^8–11^. To date, lncRNA dysregulation have been reported in numerous tumor types^11,12^, marking them as potential mediators of tumorigenicity and compelling targets for future therapeutic strategies.

LncRNAs regulate a wide variety of cellular processes, such as development and differentiation, cell cycle progression, and apoptosis^13,14^. Thus far, lncRNAs have been shown to regulate gene expression at both the transcriptional and post-transcriptional levels by a variety of mechanisms^7,15–17^. Furthermore, lncRNA regulation of gene expression can be somewhat categorized based on whether the subcellular localization is nuclear or cytoplasmic. Nuclear lncRNAs typically regulate gene expression at the chromatin level, often through DNA-RNA interactions at specific genomic loci, recruitment of epigenetic modifying complexes^18^, and regulation of nuclear organization^5,7,18,19^. In contrast, cytoplasmic lncRNAs often regulate gene expression at the post-transcriptional level through mechanisms such as control of mRNA stability, modulating translation, or serving as competing endogenous RNA “decoys” to sequester other molecules (particularly miRNAs) and prevent them from binding to target mRNAs^15,17,20^.

We previously utilized gene expression data from human tumors and adjacent normal tissues to identify lncRNAs that are dysregulated in cancer and could potentially affect tumor initiation and progression^9,10,21,22^. Other studies have also used similar strategies to identify lncRNAs that are dysregulated in human tumors^3,11,23–28^. For example, the lncRNA HOTAIR, which is highly up-regulated in metastatic breast tumors, contributes to the metastatic phenotype via interactions with the PRC2 complex^29^. Another study demonstrated that tissue-specific loss of *Xist* expression in blood progenitors leads to hematological cancers in female mice^30^. These few examples as well as many other studies have clearly demonstrated that dysregulation of lncRNAs contribute to tumorigenesis and may emerge as potential drug targets^19,31^.

In this study, we demonstrate extensive dysregulation of lncRNAs in colon cancer using RNA sequencing (RNA-seq) data from The Cancer Genome Atlas (TCGA). We identify and characterize the novel long non-coding RNA lincDUSP as a candidate oncogene in colon cancer. Knockdown studies demonstrated that depletion of lincDUSP is sufficient to abrogate the tumor phenotype, including decreased proliferation and clonogenic potential, and increased susceptibility to apoptosis. LincDUSP knockdown also results in extensive changes in gene expression, particularly for genes involved in cell cycle regulation and DNA damage response pathways. We further show that lincDUSP knockdown increases DNA damage response and perturbs cell cycle progression. These results suggest that lincDUSP and other as-yet-uncharacterized lncRNAs may play key roles in cancer initiation and progression.

## Results

### LincDUSP is a Novel lncRNA Upregulated in a Subset of Colon Tumors

**Figure 1:**
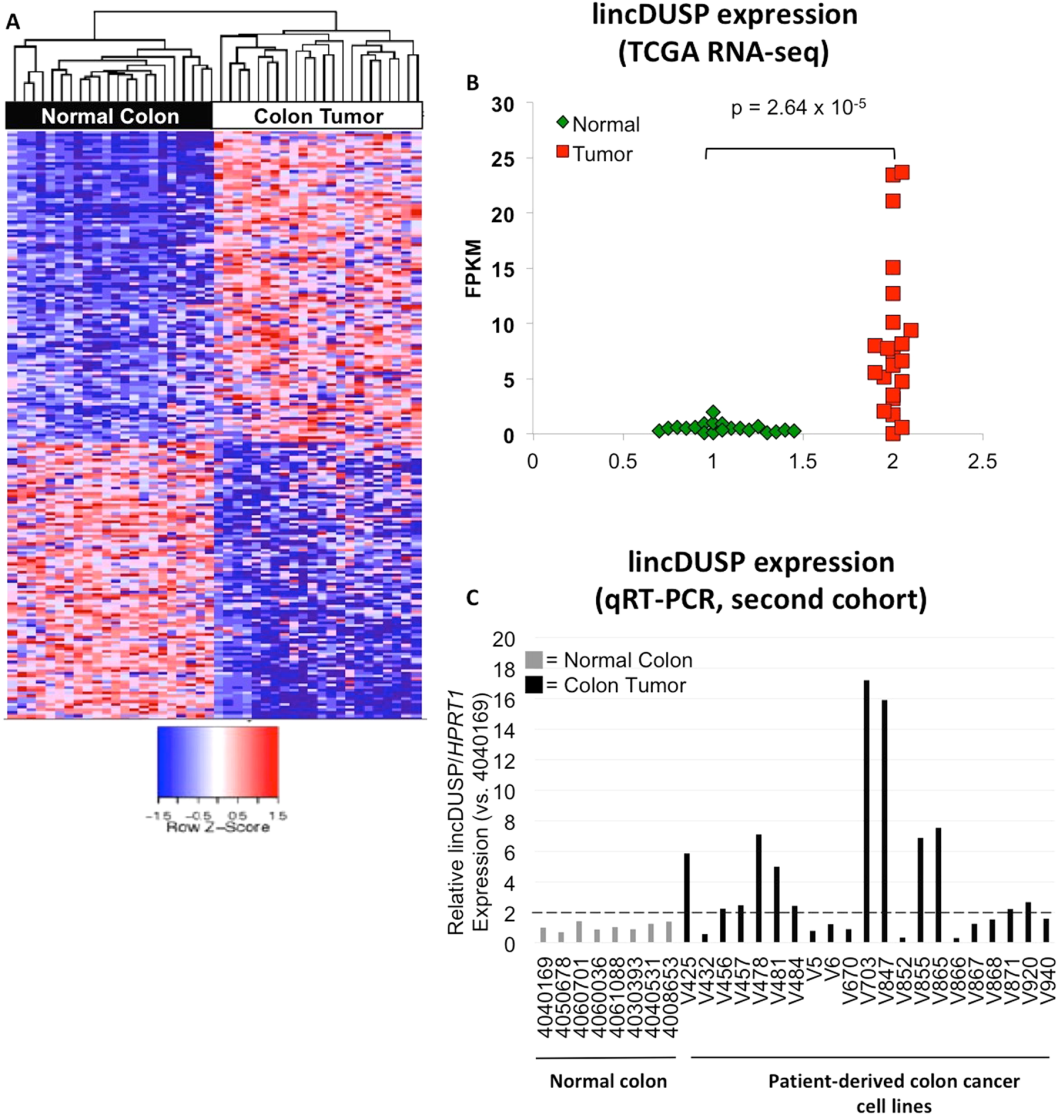
lincDUSP is a candidate oncogenic lncRNA that is overexpressed in a subset of colon tumors. (**A**) Heatmap of differentially expressed lncRNAs (>2-fold change, p < 0.05) identified from 22 colon tumors and 22 matched normal colon tissue from TCGA RNA-Seq data. (**B**) Cluster graph of lincDUSP FPKM values from tumors vs. normal colon from TCGA RNA-Seq data. (**C**) Taqman qRT-PCR for lincDUSP in an independent cohort of patient-derived normal colon and colon tumor samples. Values were normalized to the normal colon sample ID number 4040169, and *HPRT1* was used an endogenous control.

To identify novel candidate oncogenic lncRNAs in colon cancer, we leveraged publically available RNA sequencing data from The Cancer Genome Atlas (TCGA) database^32^. RNA-Seq data was obtained for 22 colon tumors and 22 matched normal colon tissue samples (Supporting Data File 1). Sequencing reads were mapped to the human genome (hg19) and annotated using a previously identified set of lncRNAs^33^. Expression levels for annotated lncRNAs were calculated using Fragments Per Kilobase per Million reads (FPKM) values, and differentially expressed lncRNAs were defined as transcripts showing >2-fold change in average FPKM values and *p*-value < 0.05 between colon tumor and matched normal tissue sample sets. Using these criteria, approximately 200 lncRNAs were identified that were differentially expressed between normal colon tissue and colon tumors (Fig. 1A, Supplemental Data File 1). The 20 lncRNAs with the highest fold change in expression in colon tumors vs. normal colon were selected as candidate oncogenic lncRNAs for further analysis. Cluster graphs were created for each of these candidates to better visualize the distribution of expression values for colon tumors and normal colon tissue, allowing for the elimination of lncRNAs with skewed average FPKM values due to outlier samples. The remaining candidate lncRNAs identified by this method included MALAT-1, a known oncogenic lncRNA involved in colon cancer^26,34^, suggesting that our approach is likely to detect novel oncogenic lncRNAs.

To ascertain that our candidate lncRNAs are upregulated in colon tumors, we next validated their expression by quantitative real-time PCR (qRT-PCR) in an independent cohort of normal colon tissues and patient-derived colon tumor cell lines. These cell lines are derived from patient tumors and are only maintained in culture for a limited number of passages to minimize genetic and epigenetic changes from the original tumors. These analyses led to the identification of a strong candidate lncRNA, lincDUSP (Gene Symbol: LINC01605, RefSeq: NR_121621.1) for functional studies. Specifically, lincDUSP expression is consistently modest in normal colon but is significantly upregulated in colon tumors in both the TCGA dataset (Fig. 1B) and our patient-derived colon tumor samples (Fig. 1C). Specifically, lincDUSP is upregulated in 20/22 tumors vs. match normal tissue examined in the TCGA dataset (Fig. 1B), and in 12/21 patient-derived tumor cell lines vs. normal colon, which is an independent cohort (Fig. 1C). Based on these observations, lincDUSP was selected for further studies as a candidate oncogenic lncRNA in colon cancer.

LincDUSP (Gene Symbol: LINC01605, RefSeq: NR_121621.1) is located at 8p11.23 and spans 4981 bp in the genome. The mature RNA consists of 3 exons totaling 528 bp. Importantly, lincDUSP was not shown to contain small ORFs based on a large-scale study of human lincRNAs^35^, providing supportive evidence that this RNA does not encode small peptides. lincDUSP is expressed in several normal tissues in humans including colon, breast, and lung (Supplemental Fig. 1). Further, subcellular RNA isolation demonstrated that lincDUSP is enriched in the nucleus (Supplemental Fig. 2), suggesting that lincDUSP may play a role in gene regulation via interaction with chromatin.

### Knockdown of lincDUSP Reduces Proliferation and Clonogenic Potential, and Potentiates Apoptosis Induction *in vitro*

To determine whether lincDUSP shows oncogenic activity in colon cancer, we decided to test the effects of knocking down lincDUSP in patient-derived colon tumor cell lines (Fig. 1C). Given that these cell lines are derived directly from colon tumors and undergo limited passaging in culture, they are predicted to better model colon cancer relative to commercially available colon cancer cell lines. Based on lincDUSP expression levels observed in our cohort of patient-derived colon cancer cell lines (Fig. 1C), we selected the two cell lines, V703 and V481, for further studies since: a. both cell lines show high endogenous expression of lincDUSP making them suitable for knock down experiments; b. these two cell lines are suitable for transfection experiments based on previous studies; c. the two cell lines share common clinical features including derivation from stage B tumors, wildtype BRAF, and mutant TGFb RII (Supplemental Fig. 3).

**Figure 2:**
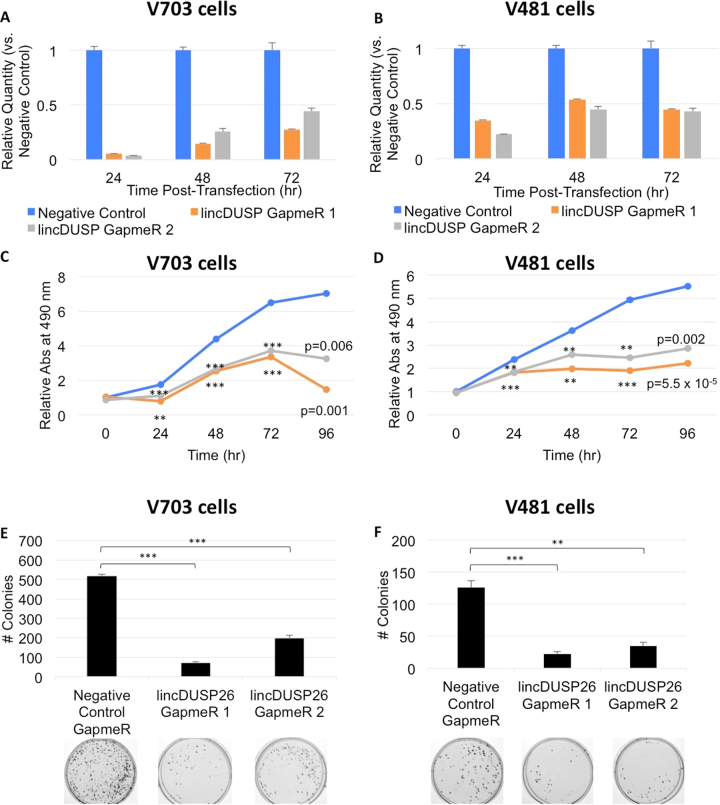
Knockdown of lincDUSP reduces proliferation and colony formation *in vitro*. (**A**,**B**) Verification of lincDUSP knockdown in V703 and V481 cells by LNA GapmeRs at 24, 48, and 72 hours. Error bars = SD (n = 2). (**C**,**D**) Proliferation assay in V703 and V481 cells treated with control GapmeRs vs. lincDUSP GapmeRs. The knockdown of lincDUSP significantly affects cell proliferations as compared to control cells. (**E**,**F**) Colony formation assay in V703 and V481 cells treated with control GapmeRs vs. lincDUSP GapmeRs. The depletion of lincDUSP has significant effects on the clonogenic capacity of colon cancer cells. Representative images shown below graph. Asterisks denote significant difference vs. negative control GapmeR by two-tailed t-test. **<0.05; ***<0.01.

We first re-confirmed the upregulation of lincDUSP expression in V703 and V481 patient-derived colon cancer cell lines (Supplemental Fig. 4). Given that Locked Nucleic Acid (LNA) antisense oligonucleotides are more effective for knockdown of nuclear-localized transcripts versus siRNA^36^, we chose Antisense LNA GapmeRs™ (Exiqon, hereafter referred to as “GapmeRs”) to knock down lincDUSP expression. Knockdown was performed by transfection of V703 and V481 cell lines with either non-targeting (Negative Control) or two distinct lincDUSP-specific GapmeRs (GapmeR-1 and GapmeR-2). This resulted in up to 90% knockdown at 24 hours post-transfections (Fig. 2A,B), and significant knock downs in both cell lines were maintained up to 72 hours post transfections (Fig. 2A,B). Following successful knockdown of lincDUSP in both cell lines, we assessed cell proliferation in cells transfected with either negative control or lincDUSP-specific GapmeRs over 96 hours. Strikingly, lincDUSP knockdown by two distinct GapmeRs showed a significant decrease in cell proliferation as early as 24 hr post-transfection in both cell lines, which was sustained throughout the time course (Fig. 2C,D). Consistent with this result, knockdown of lincDUSP also resulted in a significant decrease in colony formation, suggesting that clonogenic potential is reduced upon lincDUSP knockdown (Fig. 2E,F). These data demonstrate that lincDUSP expression is associated with increased proliferation and enhanced clonogenic potential.

**Figure 3:**
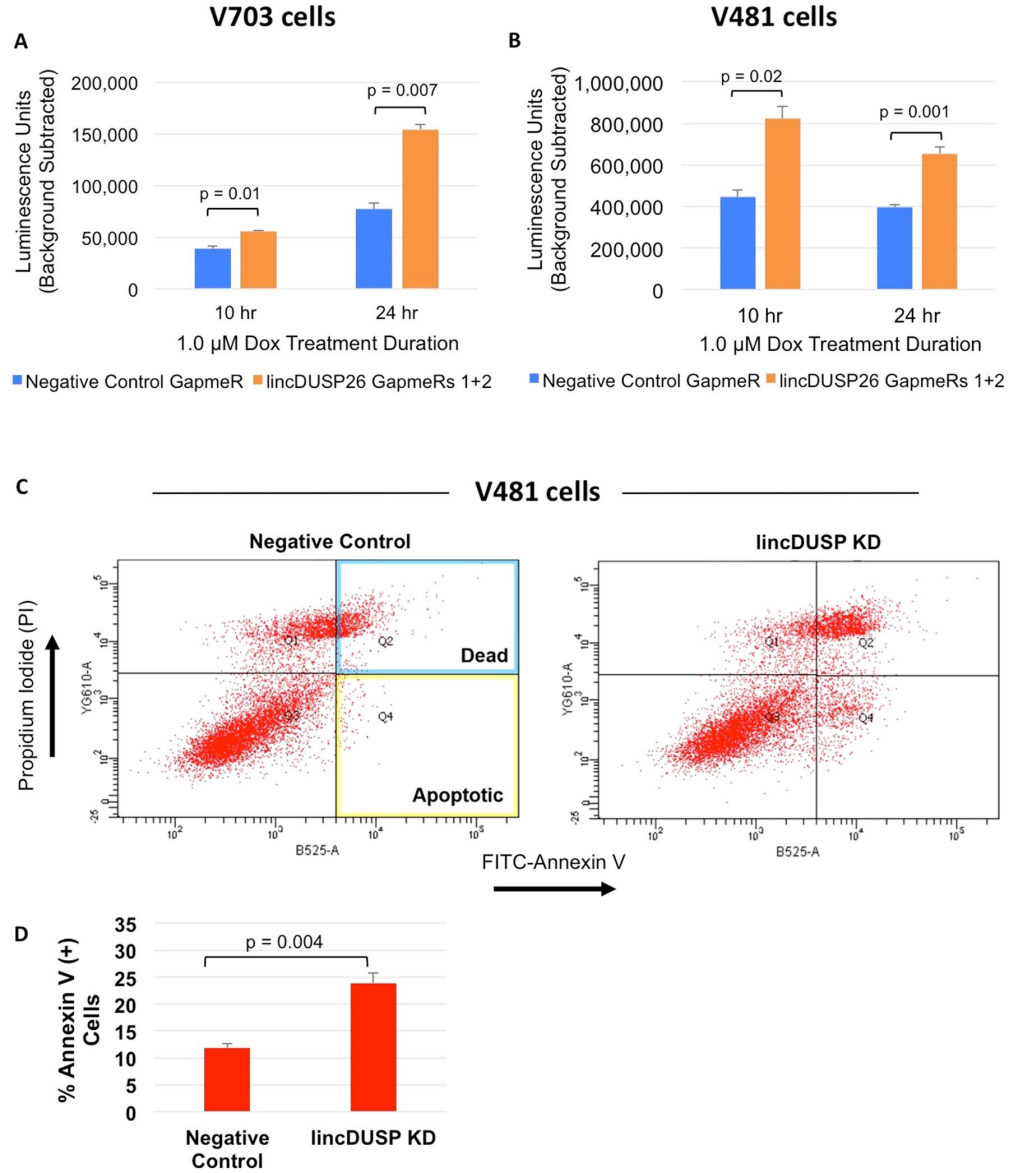
Knockdown of lincDUSP potentiates apoptosis induction *in vitro*. (**A**,**B**) Caspase 3/7 GLO Assay was performed in V703 (Panel A) and V481 (Panel B) cells treated with 1.0 uM doxorubicin (Dox) for indicated timepoints 24 hours post-transfection with indicated GapmeRs. The knock down of lincDUSP was associated with increased Caspase 3/7 activity in both cell lines. P-value generated using student’s t-test; error bars = SE (n = 3). (**C**) Flow cytometry analysis of surface Annexin V in V481 cells transfected with control or lincDUSP-specific GapmeRs. At 48 hours post-transfection, cells were treated with 2.0 µM doxorubicin for 4 hours prior to staining. (**D**) Quantitation of Annexin V flow cytometry data. P-value generated using student’s t-test; error bars = SE (n = 4 from two independent experiments).

Cancer cells demonstrate decreased susceptibility to apoptosis. Thus, we carried out assays to determine if lincDUSP expression affects colon cancer cells susceptibility to apoptosis. To that end, we performed a Caspase 3/7 activity assay in both V703 and V481 patient-derived colon cancer cells to characterize terminal apoptosis induction. Cells were treated with doxorubicin, which enhances apoptosis induction due to extensive replication-associated double stranded DNA breaks^37^, then verified that doxorubicin was an effective agent to induce apoptosis in both of our patient-derived cell lines at 10 and 24 hours post treatment (Supplemental Fig. 5A,B). We also confirmed that doxorubicin treatment did not significantly affect lincDUSP expression in either the negative control or lincDUSP-specific GapmeR-treated cells (Supplemental Fig. 6). Next, we examined the effects of knocking down lincDUSP on terminal apoptosis as described above, and found that the depletion of lincDUSP significantly increased Caspase 3/7 activity in both doxorubicin-treated V703 and V481 cells (Fig. 3A,B). These findings suggest that lincDUSP knockdown sensitizes colon cancer cells to apoptosis. We next wanted to confirm that the knock down of lincDUSP is associated with increased susceptibility to apoptosis in colon cancer cells using a second independent method. Annexin V, a membrane phospholipid, is rapidly exposed on the cell surface following apoptosis induction^38^. Knock down of lincDUSP was carried out in V481 cells for 48 hours, and subsequently, cells were treated with doxorubicin for 4 hours prior to staining and performing flow cytometry analysis (see methods). Consistent with the Caspase 3/7 results, lincDUSP knockdown in doxorubicin-treated V481 cells significantly increased populations of both dead cells (AV+/PI+) and cells actively undergoing apoptosis (AV+/PI−) (Fig. 3C). Quantification of the percentage of Annexin V positive cells is shown in Fig. 3D. A similar analysis was also performed in V703 cells, but due to a significant percentage of V703 cells that are dying at baseline, it was not possible to assess the effects of lincDUSP KD in this cell line by this method. Taken together, these results indicate that the knockdown of lincDUSP increases the susceptibility of colon cancer cells to apoptosis.

### LincDUSP Knockdown Perturbs DNA Damage Response and Cell Cycle Control Gene Pathways

**Figure 4:**
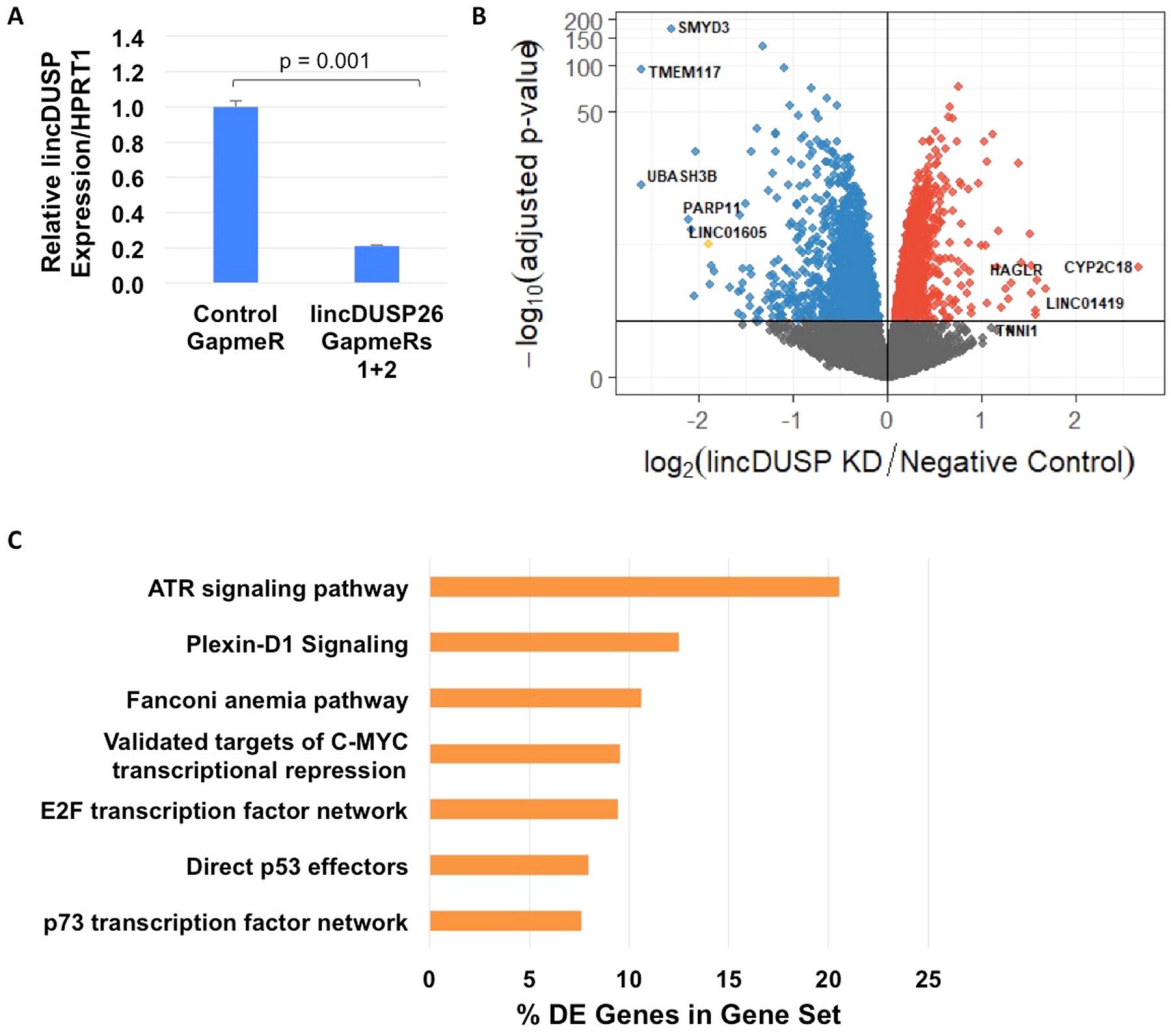
Knockdown of lincDUSP results in gene expression changes in cell cycle control pathways. (**A**) Verification of lincDUSP knock down by qRT-PCR prior to utilizing the RNA for RNA-seq. (**B**) Volcano plot of gene expression changes upon lincDUSP KD in V703 cells assayed by RNA-Seq. Genes with significantly decreased expression are shown in blue; genes with significantly increased expression are shown in red; LINC01605 (lincDUSP) shown in yellow. (**C**) Summary of significantly enriched pathways in V703 cells post lincDUSP KD (FDR < 0.05). The complete data is presented in Supporting Data File 3.

In an effort to elucidate the molecular mechanism of lincDUSP, we first performed RNA sequencing on V703 cells treated with either negative control or lincDUSP-specific GapmeRs (Fig. 4A). RNA-Seq analysis led to the identification of 797 differentially expressed genes (Fig. 4B, Supporting Data File 2). To determine whether lincDUSP regulates gene expression *in cis*, we examined this differentially expressed gene set for the five genes closest to the lincDUSP locus (*ZNF703*, *ERLIN2*, *PROSC*, *BRF2*, and *ADGRA2*). Of these genes, only *ADGRA2* was differentially expressed (+280 kb downstream of lincDUSP; −log2 fold change = −0.589, adjusted *p-*value = 4.56 × 10^−10^), suggesting that lincDUSP does not primarily regulate gene expression *in cis*. Following identification of differentially expressed genes upon lincDUSP knockdown, NCI Pathway Analysis was used to identify significantly over-represented cancer-related pathways regulated by lincDUSP. Interestingly, pathway analysis yielded several pathways related to DNA damage sensing/DNA repair (ATR signaling pathway, Fanconi anemia pathway, p53 effectors) and cell cycle regulation (E2F transcription factor network, targets of C-MYC transcriptional repression), indicating that lincDUSP may be involved in regulation of the DNA damage response and/or cell cycle progression (Fig. 4C; Supporting Data File 3).

### LincDUSP Binds Chromatin at Multiple Sites Genome-Wide Associated with Differentially Expressed Genes

To identify genes that are potentially directly regulated by lincDUSP, we next sought to determine the genome-wide chromatin binding sites for lincDUSP. Chromatin isolation by RNA purification (ChIRP) coupled to high-throughput sequencing (ChIRP-seq) is a powerful method for identifying genome-wide chromatin binding sites for lncRNAs^39^. Using five distinct biotinylated antisense oligonucleotides tiling the lincDUSP sequence, we purified lincDUSP from cross-linked cell lysates and confirmed probe specificity by determining significant enrichment of lincDUSP versus non-targeting control probes by qRT-PCR (Supplemental Fig. 7A). We then performed ChIRP-seq in V703 colon cancer cells, identifying 3913 peaks that were enriched at least 5-fold over the non-targeting control (Supporting Data File 4); importantly, a large peak overlapping the transcription start site of lincDUSP was identified, further confirming lincDUSP probe specificity (Supplemental Fig. 7B).

**Figure 5:**
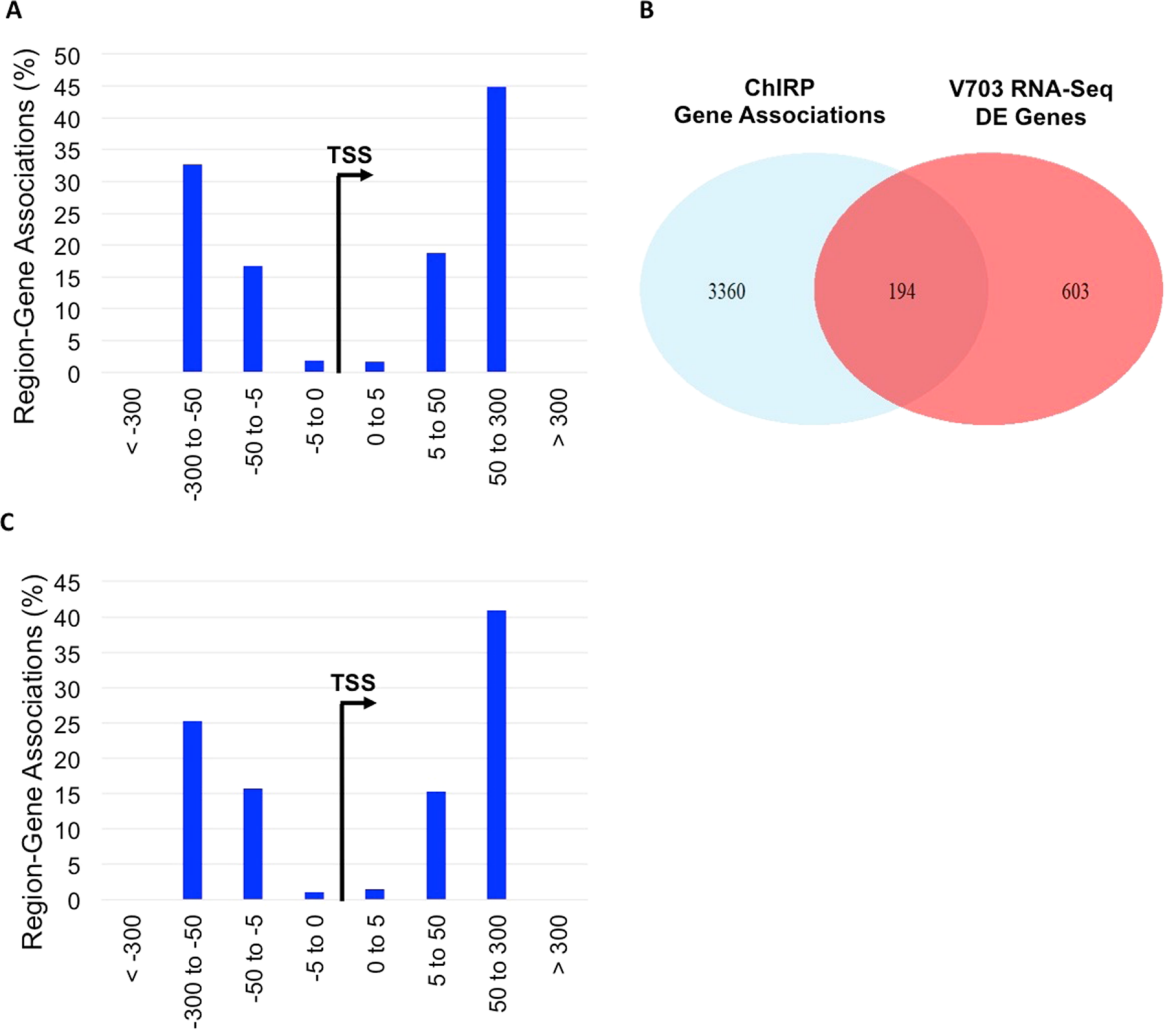
lincDUSP associates with chromatin within regulatory regions of differentially expressed genes. (**A**) The genome-wide occupancy of lincDUSP was identified by ChIRP-seq. The distribution of distances of lincDUSP ChIRP-Seq peaks to transcription start sites of protein-coding genes is shown. (**B**) Overlap between genes that are within 300 kb of a lincDUSP ChIRP-seq peak and differentially expressed genes identified in V703 cells post lincDUSP KD (RNA-Seq). (**C**) The distribution of distances of lincDUSP ChIRP-Seq peaks to transcription start sites of protein-coding genes that are also differentially expressed upon KD of lincDUSP.

Using the Genomic Regions Enrichment of Annotations Tool (GREAT)^40^, we annotated peaks associated with gene regulatory regions (5 kb upstream, 1 kb downstream) with up to 300 kb extension to capture potential long-range enhancer-like interactions. Following GREAT annotation, 4734 gene associations were identified within the ChIRP-Seq data set, including 3553 unique gene associations (Supporting Data File 4). Based on the distribution of distances from each ChIRP peak to the transcription start site of the associated gene (Fig. 5A), approximately 3.5% of peaks fell within 5 kb of the gene transcription start site (TSS), while the majority (77.5%) were greater than 50 kb upstream or downstream of the TSS. These findings could potentially indicate that lincDUSP mediate long-range chromatin looping. Notably, lincDUSP ChIRP-seq showed chromatin occupancy near *EXO1*, *PCNA*, and *RFC3*, which are known to be involved in replication-associated DNA repair^41–44^, see below.

Next, we intersected ChIRP-Seq gene associations with the differentially expressed gene set (both identified in V703 cells), yielding 261 associations with 194 unique genes (Fig. 5B; Supporting Data File 5). The distribution of distances from each ChIRP peak to the transcription start site of these genes was similar to the complete V703 ChIRP-Seq data set (Fig. 5C), with the majority of peaks (66.3%) occurring greater than 50 kb upstream or downstream of the transcription start site and few peaks (2.68%) falling within the putative promoter region (5 kb upstream +1 kb downstream of TSS)^40^. These findings may indicate that lincDUSP regulates gene expression by enabling interaction between *cis*-regulatory elements and gene promoters, as has been previously described for multiple lncRNAs^23,45,46^.

### LincDUSP Knockdown Impairs S-Phase Progression and Increases DNA Damage Response Induction *in vitro*

**Figure 6:**
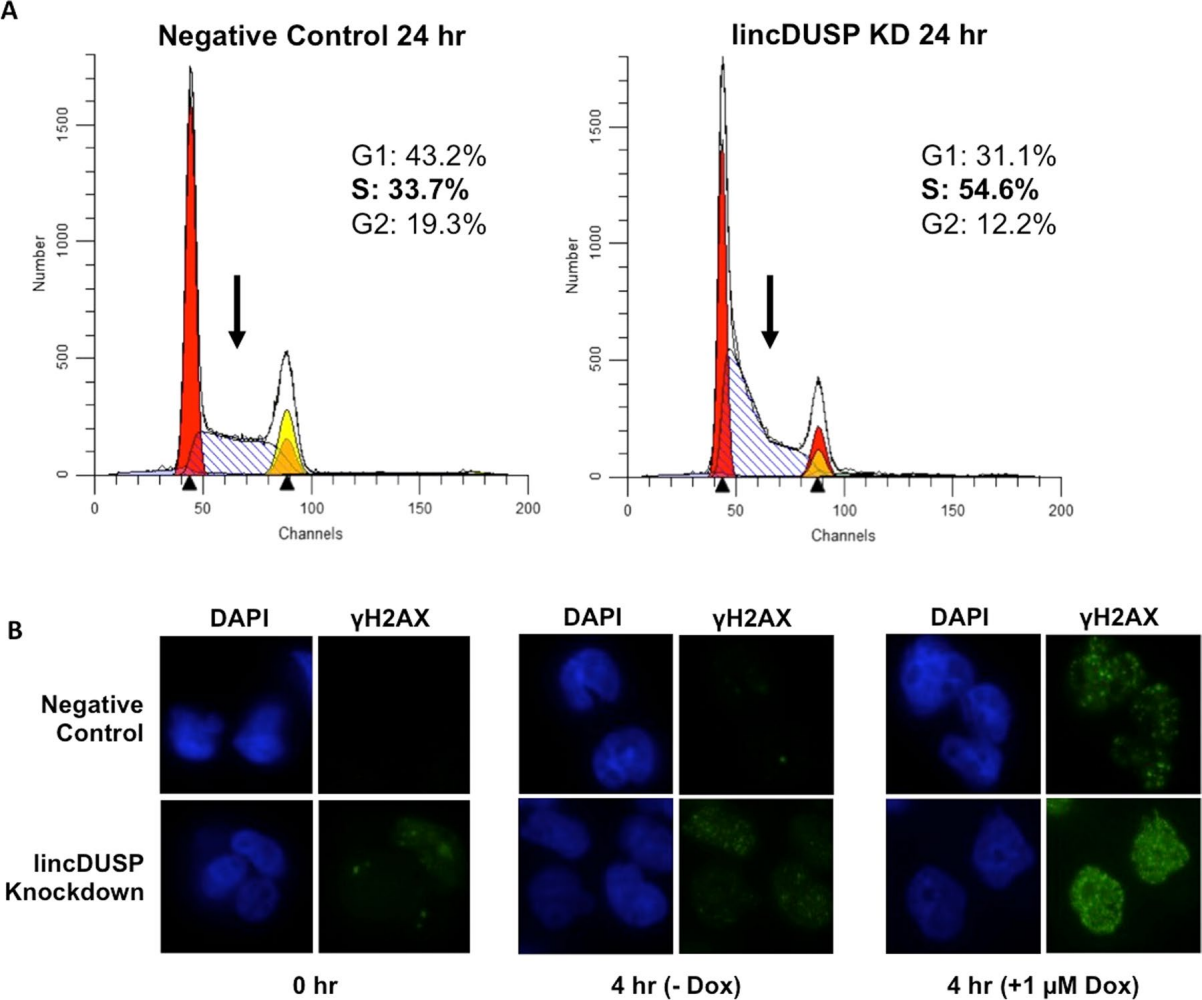
lincDUSP knockdown affects cell cycle distribution and DNA damage response induction. (**A**) Cell cycle analysis of DAPI-stained V703 cells 24 hours post-transfection with indicated GapmeRs. Left panel: Negative control GapmeRs, Right panel: lincDUSP-specific GapmeRs. Black arrows indicate S-phase peak. Cell cycle models were fit using ModFit LT v4.0. (**B**) Representative images of Alexa 488-labeled γH2AX in V481 cells. Cells were transfected with indicated GapmeRs; time 0 represents cells 48 hours post-transfection. Cells were treated with 1 µM Doxorubicin (Dox) where indicated.

Given that several DNA damage response and cell cycle control gene pathways appear to be dysregulated upon lincDUSP knockdown (Fig. 4C), and that lincDUSP binds chromatin near genes involved in replication-associated DNA repair (Fig. 5), we further investigated the effects of lincDUSP on cell cycle and DNA damage response. We first analyzed changes in the cell cycle phase distribution in DAPI-stained V703 cells upon lincDUSP knockdown. A significant increase in the early S-phase population was evident at 24 hours post-transfection with lincDUSP-specific GapmeRs versus non-targeting GapmeRs (Fig. 6A), indicating a possible lesion in cell cycle progression through S-phase. Next, we sought to characterize overall DNA damage induction in patient-derived colon tumor cells upon lincDUSP knockdown by γH2AX staining. The histone protein H2AX is rapidly phosphorylated by ATR at sites of double-stranded DNA damage or stalled replication forks, making it a useful marker of double-stranded DNA damage response induction and replicative stress^47,48^. After knockdown of lincDUSP, γH2AX foci were increased, which was further augmented after induction of DNA damage by doxorubicin treatment (Fig. 6B). Taken together, these findings provide evidence for a potential role of lincDUSP in dysregulation of the replication-associated DNA repair response in colon cancer.

## Discussion

In this study, we characterize a novel lncRNA, lincDUSP, with potential oncogenic function in colon cancer. LincDUSP expression is upregulated in a subset of colon tumors versus normal colon in two independent cohorts. Further, depletion of lincDUSP resulted in marked reduction of cell viability, increased cell death, and an increased susceptibility to apoptosis in patient-derived colon cancer cell lines. Analysis of gene expression changes and lincDUSP chromatin occupancy suggest that lincDUSP directly regulates expression of key genes involved in cell cycle progression and DNA replication-associated repair. Identification of proteins associated with lincDUSP as part of a functional ribonuclear protein complex may aid in further revealing the mechanisms of lincDUSP-mediated gene regulation.

One intriguing possibility raised by these data posits that increased apoptosis following lincDUSP knockdown in tumor cells may be secondary to failure to pass the S-phase cell cycle checkpoint. DNA replication stress, including stalled replication forks, can lead to accumulation of double strand breaks (DSBs), prompting cells to either repair the damage or induce cell death in case of catastrophic damage^49–51^. In the context of colon cancer, which is often characterized by extensive DNA damage genome-wide, suppression of the DNA damage response would provide a considerable survival advantage for cancer cells. Additional studies will be necessary to confirm involvement of lincDUSP in regulation of DNA repair response pathways, including characterization of key downstream effector genes.

Currently, the clinical and genetic factors that distinguish colon tumors with lincDUSP overexpression from other colon tumors with more modest expression are unclear; indeed, it is also unknown how lincDUSP becomes overexpressed in this context. Proposed mechanisms of lncRNA dysregulation include locus amplification (similar to HER2 amplification in breast cancer); large-scale DNA mutations such as deletions, insertions, and translocations; or smaller-scale deletions at critical functional regions within the lncRNA sequence^52^. Further exploration of these mechanisms will aid in understanding the role of lncRNA dysregulation in disease pathogenesis. In particular, identifying temporal changes in lncRNA expression along the course of disease progression will be important in deciphering how these regulatory molecules contribute to diseases such as cancer.

In summary, our current findings add to emerging studies, which demonstrate a clear involvement of lncRNAs in tumorigenesis and metastasis. These lncRNAs could be exploited as biomarkers and/or therapeutic targets in various cancers, and may result in fewer side effects due to their cell-type specific expression. Indeed, recent advances in drug development suggest that RNA molecules could emerge as direct drug targets in various human diseases. The identification of lncRNAs, such as lincDUSP, with oncogenic functions in human cancers is a key first step toward exploring the feasibility of targeting these RNAs using small molecules.

## Methods and Materials

The authors confirm that all methods and protocols were carried out in accordance with guidelines and regulations. Human cell lines used in these studies were de-identified prior to use in these studies. The cell lines were derived using an approved IRB protocol to Dr. Sanford Markowitz.

### Cell Culture

Patient-derived colon cancer cell lines V481 and V703 were maintained in MEM 2 + media (2% FBS, 2 mM glutamine, 1 ug/mL hydrocortisone, 10 ug/mL insulin, 0.86 ng/mL selenium, 2 ug/mL transferrin, 50 ug/mL gentamicin) at 37 °C, 5% CO_2_ and passaged approximately every 5 days by trypsinization. New cells were thawed approximately every 20 passages.

### RNA Isolation and cDNA Preparation

Trizol reagent (Life Technologies) was added directly to cell culture plate wells (1 mL per 1 × 10^7^ cells) and incubated at room temperature for 5 min. Phenol/chloroform RNA isolation proceeded using the QIAGEN RNeasy Mini Kit protocol with DNase digestion. RNA was quantitated using the NanoDrop 1000 Spectrophotometer. 500–1000 ng RNA was diluted to 20 μL total volume in nuclease-free H_2_O to prepare cDNA using the RNA to cDNA EcoDry™ Premix kit protocol (Clontech). cDNA was diluted to 2 ng/μL for qPCR analysis.

### Quantitative Real-Time PCR (qRT-PCR)

Taqman qPCR for lincDUSP was performed using 10 ng cDNA, 20X Taqman Probe Mixes for HPRT1 and lincDUSP (Life Technologies), and 2X Taqman Gene Expression Master Mix (Life Technologies). Samples were cycled using the standard Taqman protocol on the ABI StepOne qPCR instrument. Data was analyzed using the comparative cycle threshold (C_T_) method to obtain relative expression quantities after normalization with *HPRT1* mRNA levels. Taqman probe sequences are shown in Supplemental Table 1.

### LincDUSP Knockdown by Antisense LNA™ GapmeRs

Negative control A and lincDUSP26 Antisense LNA™ GapmeRs were purchased from Exiqon (Woburn, MA, USA). GapmeRs for lincDUSP were designed through the Exiqon design tool (http://exiqon.com/gapmer) using the TCONS_00014976 predicted RNA sequence from the UCSC Genome Browser as the input sequence. GapmeRs were initially introduced into cells using the Lipofectamine 3000 kit protocol in sufficient amounts to yield a final GapmeR concentration of 50 nM. For proliferation assays and colony formation assays, cells were “spiked” with additional GapmeRs every 48–72 hours to maintain long-term knockdown efficacy. Knockdown efficiency was assessed using Taqman qPCR for lincDUSP. GapmeR sequences are shown in Supplemental Table 2.

### Proliferation Assay

Cell proliferation was assessed using the Promega CellTiter 96® AQ_ueous_ One Solution Cell Proliferation Assay kit protocol. Cells were reverse-transfected with 50 nM negative control or lincDUSP-specific GapmeRs using the Lipofectamine 3000 protocol (described above) and plated in quadruplicate at a density of 2,000–5,000 cells/well in 5 separate 96-well plates. Cells were allowed to settle 5–6 hours before taking initial reading (0 hr); media was changed on remaining plates after 6 hours. Readings were taken every 24 hours for a total of 96 hours by adding 20 uL CellTiter 96® AQ_ueous_ One Solution to each well, incubating at 37 °C for 1 hr, and measuring absorbance at 490 nm on the Wallac Victor2 1420 Multilabel Counter.

### Colony Formation Assay

Cells were reverse-transfected with 50 nM negative control or lincDUSP-specific GapmeRs using the Lipofectamine 3000 protocol (described above) and plated in triplicate in 60 mm culture dishes at a density of 2,000–5,000 cells/plate. Colonies were allowed to grow over 14–16 days; media was spiked with 50 nM GapmeRs every 72 hours to maintain knockdown efficiency. Colonies were fixed in 10% acetic acid/10% methanol and stained with 0.5% crystal violet in methanol. Colonies were counted using the ImageJ cell counter plugin.

### Caspase 3/7 Activity Assay for Apoptosis

Cells were reverse-transfected with 50 nM negative control or combined lincDUSP-specific GapmeRs using the Lipofectamine 3000 protocol (described above), plated in quadruplicate on two separate 96-well plates at a density of 5,000–10,000 cells/well, and allowed to incubate overnight (~16 hours). Untransfected cells were plated in two sets of triplicates for DOX(+) and DOX(−) control to verify apoptosis induction. 100 uM doxorubicin (DOX) stock solution (prepared from doxorubicin hydrochloride; Fisher Scientific, Cat #ICN15910101) was diluted to 1 uM in media and added to each test well and DOX(+) control wells; 100 uL fresh media was added to DOX (−) control wells. At 10 hr and 24 hr DOX treatment, 100 uL Caspase 3/7 GLO reagent (Promega Caspase 3/7 GLO Assay) was added to each test well, incubated at room temperature for 15 min, and read on the Wallac Victor2 1420 Multilabel Counter using the luminometer setting.

### Annexin V Staining for Apoptosis

Cells were reverse-transfected with 50 nM negative control or combined lincDUSP-specific GapmeRs using the Lipofectamine 3000 protocol (described above) and plated in duplicate in 6-well culture plates at a density of 500,000 cells/well. Cells were treated with 2 uM doxorubicin (DOX) solution for 4 hours starting at 48 hours post-transfection. Cells were harvested on ice using a cell lifter, pelleted, and washed in cold PBS. Cells were processed using the FITC Annexin V Apoptosis Detection Kit I (BD Biosciences Cat #556547) and analyzed on the BD LSR II instrument using the B525 channel (FITC-Annexin V) and the YG610 channel (Propidium Iodide).

### RNA Sequencing

V703 Cells were reverse-transfected with negative control GapmeR or combined lincDUSP-specific GapmeRs using the Lipofectamine 3000 protocol (described above) and plated in triplicate at a density of 500k cells/well in a 12-well culture plate. RNA was isolated from each well 48 hours post transfection as described above. Knockdown efficiency was verified using Taqman qPCR for lincDUSP. Total RNA was processed using the TrueSeq Stranded Total RNA Library Prep Kit (Illumina RS-122–2203) and sequenced on the HiSeq. 2500 using a Paired-end Rapid Run v2 flowcell. Raw reads were aligned to the genome (hg38) using bowtie2 (v2.2.9) and tophat (v2.1.1) with gene annotations obtained from the Illumina iGenome collection. A counts table with the number of reads for each gene was then generated using samtools (v1.3) and HTSeq (v0.6.1) using the HTSeq counting option “union”. Differential gene expression analysis was performed using DESeq. 2 (v1.12.2).

### Chromatin Isolation by RNA Purification (ChIRP)

LincDUSP-specific tiling biotinylated oligonucleotides were designed from the mature lincDUSP sequence using the Biosearch Technologies ChIRP Probe Designer tool (v 4.1) (https://www.biosearchtech.com/chirpdesigner/). Probes were ordered from IDT using 5′-Biotin-TEG and phosphorothioate bond modifications; sequences are shown in Supplementary Table 3. ChIRP was performed for V703 cells as previously described^22^ using 500 pmol non-targeting or lincDUSP-specific probes/25 million cells; probes were immunoprecipitated using Dynabeads™ M-280 Streptavidin (Thermo Fisher Scientific, Cat #11205D). 50 uL beads were removed from each sample for RNA elution to confirm lincDUSP enrichment in lincDUSP pulldown.

For ChIRP-Seq, DNA was eluted from streptavidin beads by reverse-crosslinking overnight at 65 °C followed by DNA extraction with the QIAGEN DNeasy Blood and Tissue Kit (Cat #69504). Sequencing libraries with indexed adapters were prepared using the ThruPLEX® DNA-seq Kit 6 S (Rubicon Genetics Cat # R400523). Libraries were sequenced on the Illumina HiSeq. 2500 DNA Sequencer platform on a single v2 Rapid Run flowcell. Raw reads were aligned to hg38 using Bowtie2. Peaks enriched upon lincDUSP pulldown vs. non-targeting control were called using MACS software (Version 1.4.0rc2) and filtered for peaks with greater than 5-fold enrichment over non-targeting control. Peak positions were converted to hg19 using the UCSC liftOver Tool (https://genome.ucsc.edu/cgi-bin/hgLiftOver). Genomic occupancy peaks were associated with genes within 300 kb using the Genomic Regions Enrichment of Annotations Tool (GREAT)^40^.

### Pathway Analysis

Significantly enriched pathways in V703 RNA-Seq differentially expressed genes were identified using the NCI Pathway Interaction Database (hypergeometric P-value < 0.05, FDR < 0.20).

### Cell Cycle Analysis

Cells were reverse-transfected with 50 nM Negative Control or combined lincDUSP26-specific GapmeRs using the Lipofectamine 3000 protocol (described above) and plated in triplicate in 6-well culture plates at a density of 700,000–900,000 cells/well. Cells were harvested by trypsinization after appropriate timepoints (24, 48 hrs); triplicate wells were combined into one tube. Cells were washed 2× in cold PBS and fixed in ice cold 80% EtOH, then incubated on ice at least 30 min. Cells were washed 2×, then resuspended in 1 mL 1 ug/uL DAPI + 0.1% Triton X-100 in PBS and incubated at least 30 min at RT. Cells were analyzed for DNA content using the 440 nm laser on the BD LSR II cytometer. Cell cycle modeling was performed using WinList 3D (v8.0) and ModFit LT (v4.0).

### γH2AX Immunofluorescence

V481 cells were plated on #1.5 thickness round coverglasses at a density of 20,000 cells/well. After 24 hours, cells were treated with Negative Control or lincDUSP-specific GapmeRs as described above. 48 hours post-transfection, cells were treated with 2 uM doxorubicin for indicated timepoints. Cells were fixed in paraformaldehyde (3.7% formaldehyde, 0.1% Triton-X, 1 × PBS) for 30 min at 37 °C followed by 1:1 acetone:methanol for 10 min at −20 °C. Coverglasses were blocked in 1% BSA/PBS at 37 °C for 30 min before incubating in 1:200 Rabbit anti-phospo-γH2AX (Cell Signaling Technology 9718 S) at 4 °C overnight. 1:500 Goat anti-Rabbit IgG AlexaFluor 488-conjugated secondary antibody (Thermo Fisher Scientific, Cat #A-11008) was used to stain cells with 1:1000 DAPI counterstain. Cells were imaged using the Leica DM600 at 100× magnification.

### Data availability

The authors agree to share all raw and processed data. The raw data is deposited in GEO: GSE101342 and GSE112602. Processed data is provided in supporting data files and will be available on the journal website.

## Electronic supplementary material

Supplementary Information

Supplementary Dataset 1

Supplementary Dataset 2

Supplementary Dataset 3

Supplementary Dataset 4

Supplementary Dataset 5
