# Supplementary material for: Colon Cancer-Upregulated Long Non-Coding RNA lincDUSP Regulates Cell Cycle Genes and Potentiates Resistance to Apoptosis
Source: Sci Rep. 2018 May 9;8:7324. doi: 10.1038/s41598-018-25530-5 (PMC5943353; doi:10.1038/s41598-018-25530-5)
Supplement: Supplementary file 1 — Supplementary Information [file 41598_2018_25530_MOESM1_ESM.pdf]

Colorectal Cancer-Upregulated Long Non-Coding RNA lincDUSP Regulates Cell Cycle Genes and Potentiates Resistance to Apoptosis

Megan E. Forrest[1], Alina Saiakhova[1], Lydia Beard[2], David A. Buchner[1,3], Peter C. Scacheri[1], Thomas LaFramboise[1,2], Sanford Markowitz[1,2], and Ahmad M. Khalil[1,2,3*]

1. Department of Genetics and Genome Sciences 2. Case Comprehensive Cancer Center, 3. Department of Biochemistry, Case Western Reserve University, Cleveland OH

***Correspondence should be addressed to Dr. Ahmad Khalil to Dr.Ahmad.Khalil@gmail.com**

Supplemental Figure Legend:

Supplemental Figure 1: lincDUSP is expressed in several normal human tissue types. LincDUSP expression is examined in a panel of purified RNA from normal tissues (Ambion) by qRT-PCR (Taqman assays). Values were normalized to liver sample.

Supplemental Figure 2: lincDUSP is enriched in the nucleus in colon cancer cells. lincDUSP expression was assessed via Taqman qRT-PCR in cytoplasmic and nuclear fractions obtained from V703 cells using the Active Motif RNA Subcellular Isolation Kit (Cat #25501).

Supplemental Figure 3: Additional information about the two key patient-derived cell lines used in assays in the manuscript.

Supplemental Figure 4: Second validation of lincDUSP expression in the two cell lines used in functional studies. lincDUSP expression is increased in the patient-derived colon tumor cell lines selected for knockdown studies (V703 and V481). Expression was assessed using Taqman qRT-PCR for lincDUSP and HPRT1 as an endogenous control.

Supplemental Figure 5: Doxorubicin (DOX) (concentration = 1.0 µM) is sufficient to induce apoptosis in patient-derived colon cancer cell lines. Caspase 3/7 GLO assay in V703 cells (panel A) and V481 cells (panel B) treated with DOX for indicated time points. P-values obtained from student's t-test. Error bars represent SE, n=3.

Supplemental Figure 6: Doxorubicin (DOX) (concentration = 1.0 µM) has no effect on lincDUSP expression. Taqman qRT-PCR for lincDUSP expression in V703 cells treated with indicated conditions below each bar. Values were normalized to Negative control Dox (-) sample. These data demonstrate that DOX does not affect lincDUSP expression (compare bar 1 to bar 3, and bar 2 to bar 4).

Supplemental Figure 7: lincDUSP was successfully enriched by ChIRP. A) Relative quantity of lincDUSP in lincDUSP-specific ChIRP probe pull-down versus non-targeting controls, as assessed by Taqman qRT-PCR. B) ChIRP probes successfully recovered nascent lincDUSP RNA. A large lincDUSP genomic occupancy peak (indicated by red box) was identified near the 5'-end of the known lincDUSP genomic location (lincDUSP genomic coordinates: hg38 chr8:37,516,406-37,521,386; ChIRP peak coordinates: hg38 chr8:37,515,825-37,517,541). Track generated using V703 ChIRP-Seq data (lincDUSP/control) MACS peak output in Integrated Genome Browser v.9.0.0

Supplemental Figure 1

lincDUSP expression in a panel of normal human tissues

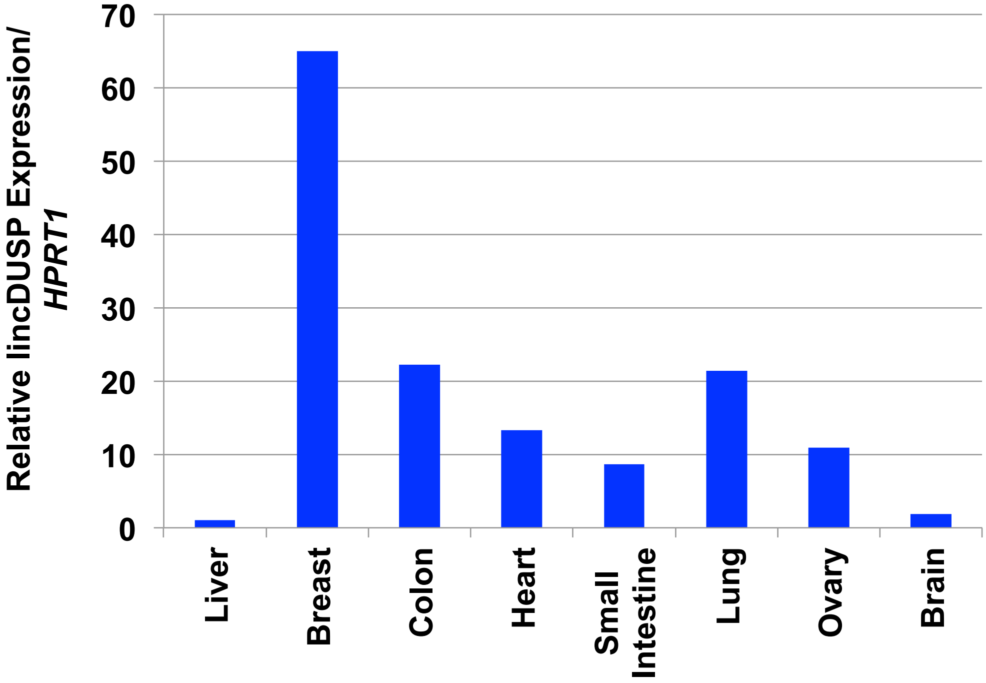

Supplemental Figure 2

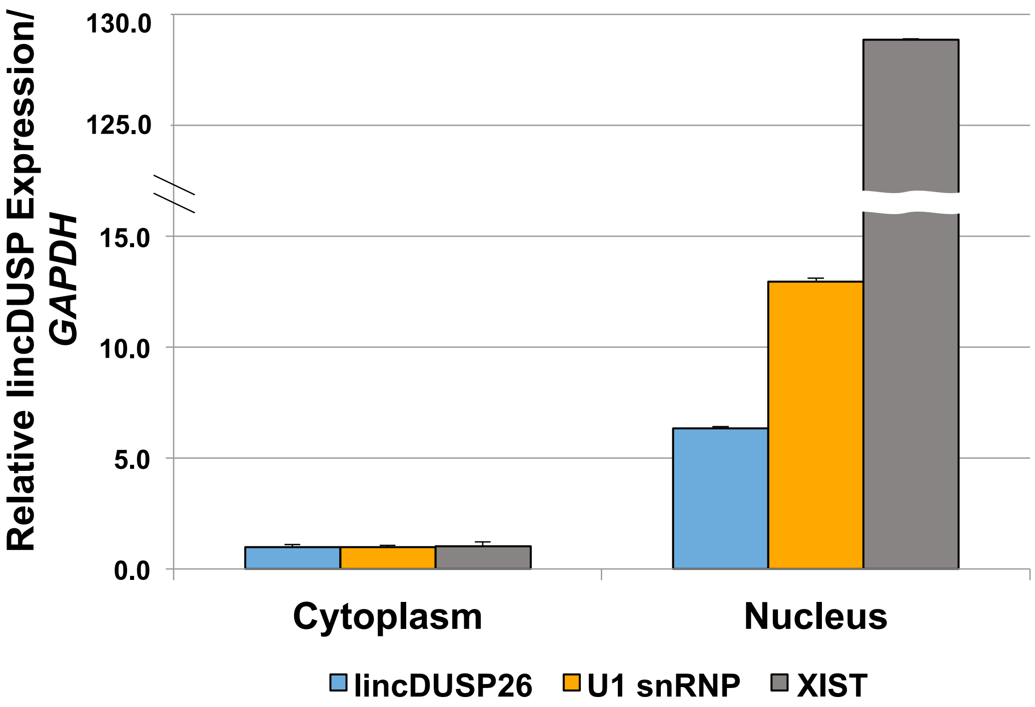

Supplemental Figure 3

Cell line	Stage	Gender	Sample Description	Detailed Stage	Primary site in colon	Side	Age at diag	BRAF (V600E)	TGFb RII mutation
V481	B	F	colon cell line	RB2	right colon	R	72	WT	MUTANT
V703	B	F	colon cell line	RB2	transverse	R	75	WT	MUTANT

Supplemental Figure 4

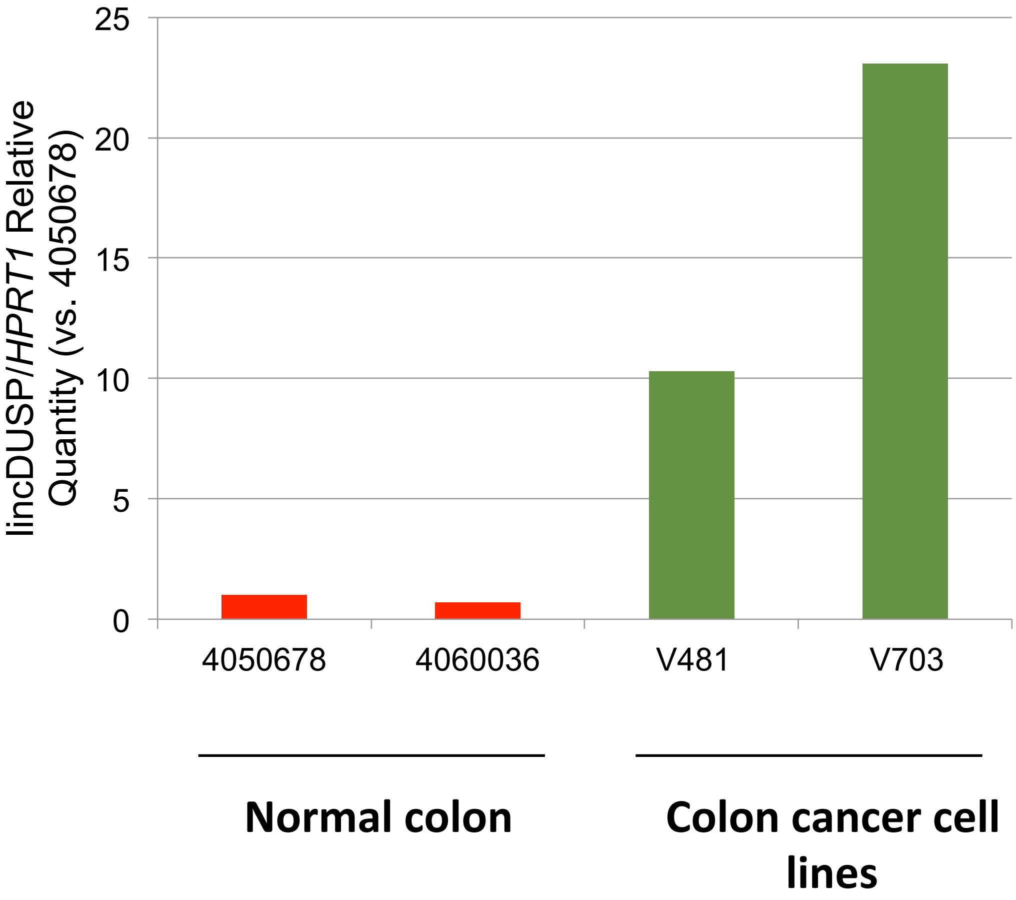

Supplemental Figure 5

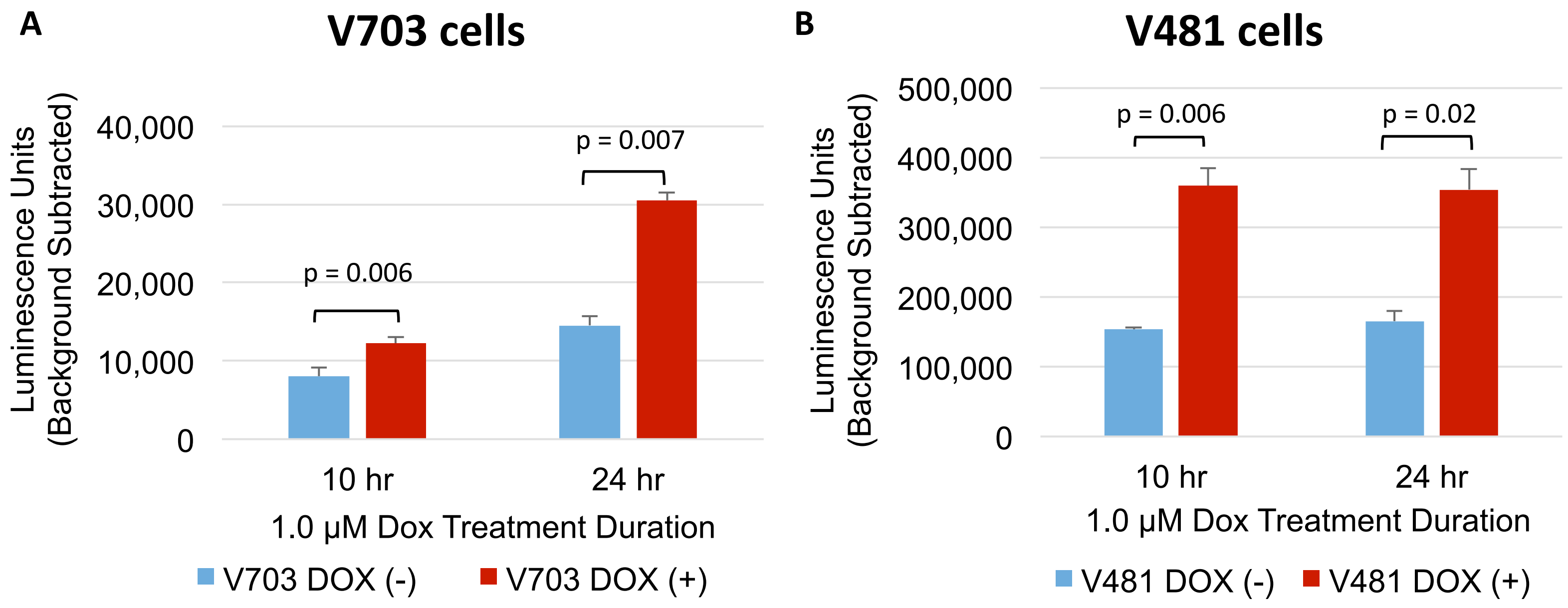

Supplemental Figure 6

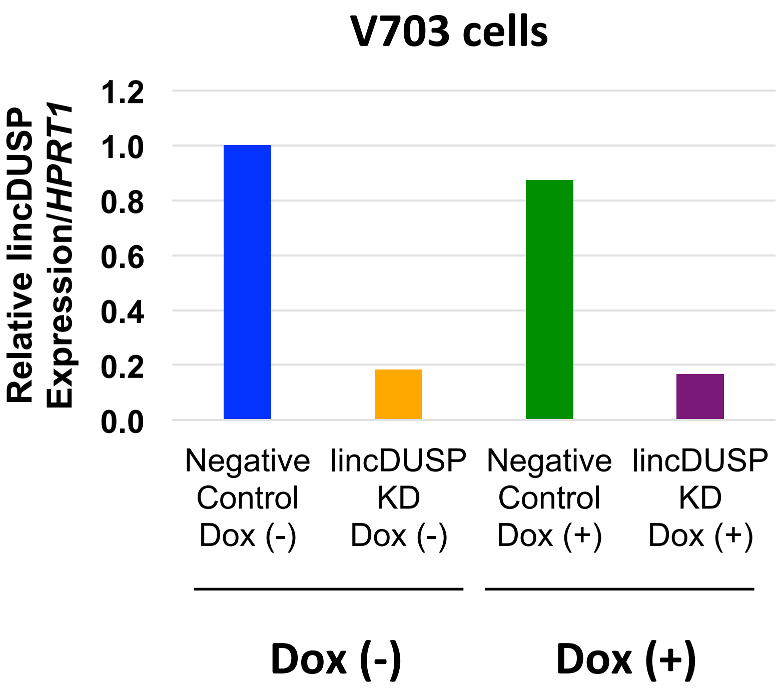

Supplemental Figure 7

A

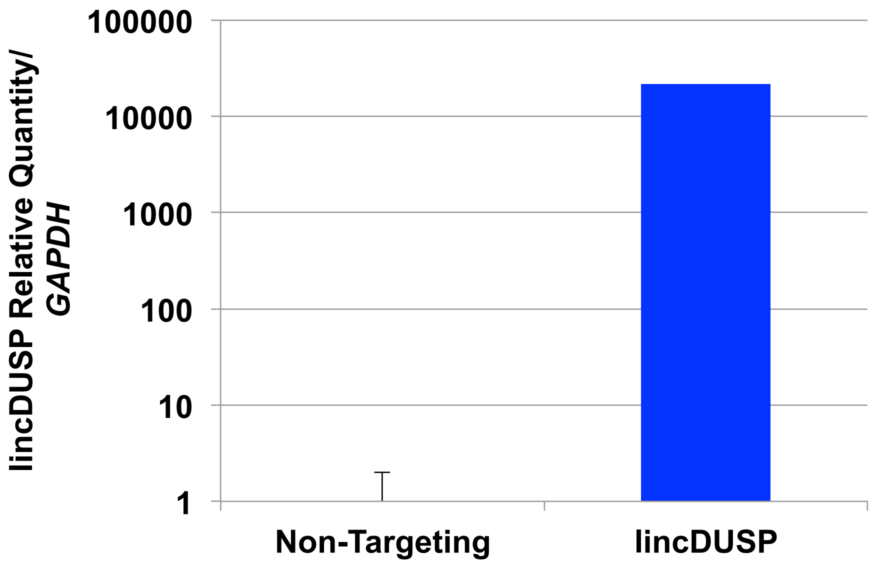

B

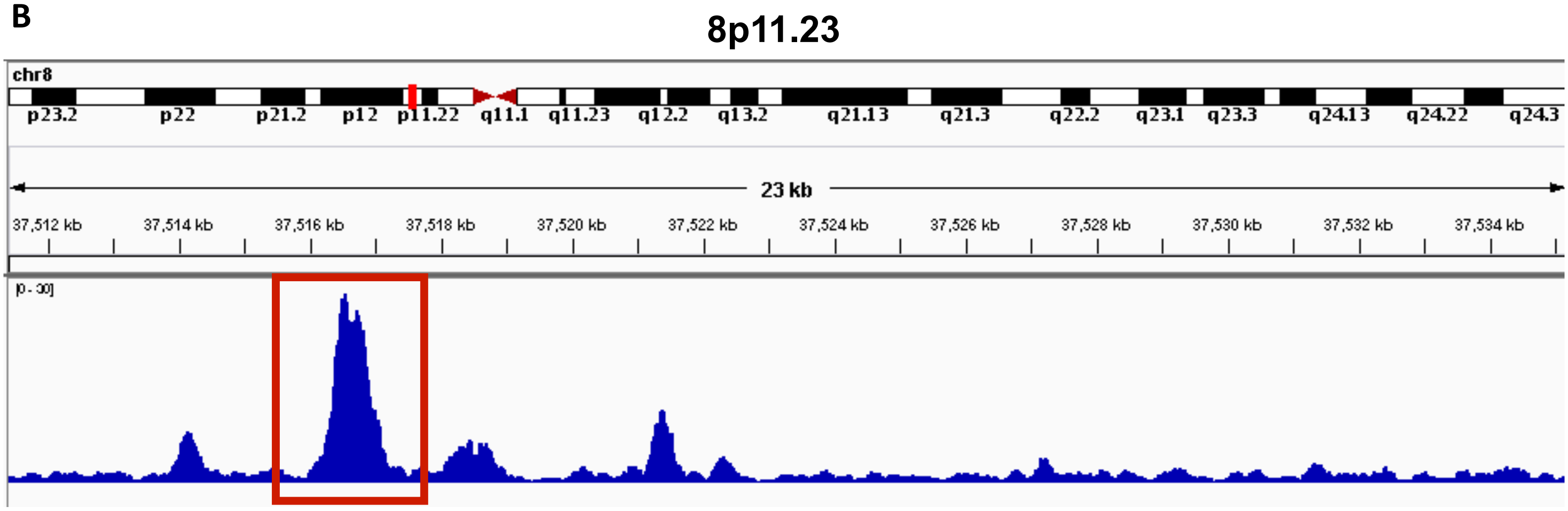

Supporting Data File Summary

Supporting Data File 1:

Differentially expressed lincRNAs identified from colon cancer TCGA RNA-Seq data.

Supporting Data File 2:

Differentially expressed mRNAs identified by RNA-Seq in V703 cells upon lincDUSP knockdown

Supporting Data File 3:

Pathway analysis of differentially expressed genes identified post lincDUSP KD

Supporting Data File 4:

Tab 1) MACS output summary of genomic occupancy peaks identified by lincDUSP ChIRP-Seq (greater than 5-fold enrichment over control probes) (hg38),

Tab 2) Conversion of peaks from hg38 → hg19

Tab 3) Summary of genomic peak-gene associations identified using GREAT.

Supporting Data File 5:

Intersection of ChIRP-Seq peak-gene associations and V703 lincDUSP KD RNA-Seq data.

Supplemental Tables

Supplemental Table 1: Taqman Probe Sequences

Gene Target	Taqman Assay ID	Probe Sequence
HPRT1	Hs03929096_g1	GCCATCACATTGTAGCCCTCTGTGTGCTCAAGG GGGGCTATAAATTCTTTGCTGACCTGCTGGA
lincDUSP	Hs04406733_g1	CAGATGGGAATGACCTGGAATCTAACACTCTAC AATTCAGACACACCACTGTCCACTTGTCCCTCC CTTCCTGCCACTGGCCCTGCCAGCGT

Supplemental Table 2: lincDUSP GapmeR Sequences

GapmeR Name	Sequence
Negative Control GapmeR A	AACACGTCTATACGC
LincDUSP GapmeR 1	CGTCTGTCAGTCATTA
LincDUSP GapmeR 2	CTGCTCAATTCCATCA

Supplemental Table 3: ChIRP-seq Probe Sequences

Probe #	Sequence	Position
Negative control-1	/5BiotinTEG/GATGTACTGGCTGGA	N/A
Negative control -2	/5BiotinTEG/GGAGTGAAGAGTGC	N/A
LincDUSP-1	/5BiotinTEG/GGAATCTGGACTTTCCATTC	42
LincDUSP-2	/5BiotinTEG/CACATCTGGTTCTTGGAAGT	153
LincDUSP-3	/5BiotinTEG/CAGTGGTGTGTCTGAATTGT	268
LincDUSP-4	/5BiotinTEG/ATCGTCTGTCAGTCATTACA	368
LincDUSP-5	/5BiotinTEG/TCCCAAGAGCCAATTTATTT	505
